# Ensemble Modeling of the Baltic Sea Ecosystem to Provide Scenarios for Management

**DOI:** 10.1007/s13280-013-0475-6

**Published:** 2014-01-12

**Authors:** H. E. Markus Meier, Helén C. Andersson, Berit Arheimer, Chantal Donnelly, Kari Eilola, Bo G. Gustafsson, Lech Kotwicki, Tina-Simone Neset, Susa Niiranen, Joanna Piwowarczyk, Oleg P. Savchuk, Frederik Schenk, Jan Marcin Węsławski, Eduardo Zorita

**Affiliations:** 1 Swedish Meteorological and Hydrological Institute, 601 76 Norrköping, Sweden; 2 Swedish Meteorological and Hydrological Institute, 426 71 Västra Frölunda, Sweden; 3 Baltic Nest Institute, Stockholm University, 106 91 Stockholm, Sweden; 4 Department of Marine Ecology, Institute of Oceanology, Polish Academy of Sciences, 55 Powstancow Warszawy Street, 81-712 Sopot, Poland; 5 Centre for Climate Science and Policy Research, Department of Water and Environmental Studies, Linköping University, 601 74 Norrköping, Sweden; 6 Stockholm Resilience Centre, Stockholm University, 106 91 Stockholm, Sweden; 7 Institute of Coastal Research, Helmholtz-Zentrum Geesthacht, Max-Planck-Str. 1, 21481 Geesthacht, Germany; 8 Institute of Coastal Research, Helmholtz-Zentrum Geesthacht, 21502 Geeshacht, Germany

**Keywords:** Baltic Sea, Ecosystem approach to management, Baltic Sea Action Plan, Climate change, Eutrophication, Multi-model ensemble approach

## Abstract

We present a multi-model ensemble study for the Baltic Sea, and investigate the combined impact of changing climate, external nutrient supply, and fisheries on the marine ecosystem. The applied regional climate system model contains state-of-the-art component models for the atmosphere, sea ice, ocean, land surface, terrestrial and marine biogeochemistry, and marine food-web. Time-dependent scenario simulations for the period 1960–2100 are performed and uncertainties of future projections are estimated. In addition, reconstructions since 1850 are carried out to evaluate the models sensitivity to external stressors on long time scales. Information from scenario simulations are used to support decision-makers and stakeholders and to raise awareness of climate change, environmental problems, and possible abatement strategies among the general public using geovisualization. It is concluded that the study results are relevant for the Baltic Sea Action Plan of the Helsinki Commission.

## Introduction

To improve the status of the Baltic Sea environment, the Helsinki Commission (HELCOM) created the Baltic Sea Action Plan (BSAP) by consistent application of the ecosystem approach to management (Backer et al. 2010). In 2007, the BSAP led to international decisions on nutrient load reductions. Policy instruments—like the Marine Strategy Framework Directive, national environmental objectives, and HELCOM’s BSAP—do not take the impact of climate change into consideration. For example, Maximum Allowable Inputs (MAIs) of the BSAP are calculated under the assumption that Baltic Sea environmental conditions are in a steady-state, biogeochemically as well as physically, and that it will reach a new biogeochemical steady-state after the internal sinks and sources have adapted to the new loads under the present, prevailing physical steady-state. Within a changing climate this assumption will not hold as the physical environment will change leading to feedbacks upon the biogeochemical cycling, e.g., by enhancing growth rates and mineralization.

Hence, the interdisciplinary ECOSUPPORT project (Advanced modeling tool for scenarios of the Baltic Sea ECOsystem to SUPPORT decision-making) running during 2009–2011 was designed to provide scientifically sound knowledge on how the combination of climate change and nutrient loads from the catchment will impact the marine ecosystem.^1^ Since climate change is likely to affect the implementation of policies and environmental objectives, the main aim of ECOSUPPORT was to provide easy access to modeled scenarios of the marine ecosystem, in order to raise awareness of stakeholders and the general public of the impacts of human activities on the ecosystem, as well as for policy decision support.

For this purpose appropriate models need to be developed. Global Earth System Models (ESMs) are fundamental tools to assess future climate change of the twenty-first century (e.g., Solomon et al. 2007). For the upcoming Intergovernmental Panel on Climate Change (IPCC) Fifth Assessment Report, a new generation of ESMs from CMIP5^2^ that include complex interactions within the Earth system, e.g., by taking feedbacks of the carbon cycle into account, was used. However, despite their complexity and great advances over recent decades, ESMs remain simplified representations of the real Earth system and are associated with a number of errors that reduce their reliability (e.g., Knutti and Sedláček 2012). One of the shortcomings of ESMs is their computational demand that limits the possibility to resolve small spatial scales. However, this information is exactly what is needed by decision-makers (e.g., BACC Author Team 2008). To bridge the gap between global model results and the regional to local information needed for impact studies, regional climate models (RCMs) have been developed as a complementary tool to ESMs allowing increased horizontal resolution and a greater number of explicitly resolved processes (Rummukainen 2010). In the dynamical downscaling approach RCMs are driven with data from ESMs at their lateral boundaries (Räisänen et al. 2004).

To be able to calculate the impact of changing climate on the Baltic Sea ecosystem, so-called Regional Climate System Models (RCSMs) are needed, comprising all relevant components of the Earth system, like atmosphere, sea ice, ocean, land surface physics, atmospheric chemistry, terrestrial and marine biogeochemistry, and marine food-web. Overall, the number of RCSM applications is still very limited. The few world-wide existing regional atmosphere–ice–ocean models are usually not coupled to terrestrial or marine ecosystem model components (Döscher et al. 2002, 2010 and references therein). Further, climate change impact studies for regional seas are very often performed using either results from a regional atmosphere model with sea surface boundary conditions from a global model as driver (Madsen 2009) or atmospheric and hydrological model outputs from global climate models directly (Lasram et al. 2010). Both approaches have significant shortcomings because sea surface boundary conditions taken from global models have significant biases at regional scale (Meier et al. 2011a).

Within ECOSUPPORT, the first steps toward a more complex RCSM for the Baltic Sea region were taken. ECOSUPPORT improved the quality of RCSM scenario simulations by (1) developing new component models with increased spatial resolution and refined process descriptions; (2) coupling model components together to study feedback mechanisms within the Earth system comprising the atmosphere–ice–ocean–land surface continuum; (3) applying a holistic, multi-stressor approach that takes the impacts of changing climate, eutrophication, and overfishing into account; (4) assessing long-term changes during 1850–2100 including the transition from oligotrophic to eutrophied states of the Baltic Sea environment; and (5) estimating uncertainties of future projections by applying a multi-model ensemble approach. Finally, ECOSUPPORT disseminated the results from scenario simulations to stakeholders and the public in a more efficient way compared to traditional dissemination techniques by using a novel visualization and communication approach. This paper summarizes major findings of ECOSUPPORT and the impacts on policies.

## Materials and Methods

### Toward Regional Climate System Modeling

Within ECOSUPPORT a hierarchy of regional models was used to downscale results from two General Circulation Models (GCMs) to the spatial scale of the Baltic Sea region including a coupled atmosphere–ice–ocean–land surface model (Döscher et al. 2002; Meier et al. 2011a), two hydrological models of differing complexity (Arheimer et al. 2012; Meier et al. 2012a), one atmospheric chemistry and transport model (Langner et al. 2009), three marine physical–biogeochemical models (Neumann et al. 2002; Eilola et al. 2009; Savchuk et al. 2012), one food-web model for the central Baltic Sea (Niiranen et al. 2012), various statistical fish population models (MacKenzie et al. 2012), and various regional to local scale models of differing parts of the Earth system for the Gulf of Finland, Vistula Lagoon, and Polish Coastal waters, for example, Biological Envelope Modeling (Weslawski et al. 2013; see also Meier et al. 2012c).

An important aspect of the ECOSUPPORT model hierarchy is the proper consideration of the land-sea continuum. ECOSUPPORT introduced for the first time the ability to simulate integrated discharge and nutrients at high-resolution for the entire Baltic Sea catchment using the Balt-HYPE model (Arheimer et al. 2012). The Balt-HYPE model was used to simulate the effects of future climate change and the interaction of climate change with a number of simpler remedial nutrient scenarios. Previously available estimates of the impacts of future climate change to discharge to the Baltic Sea were based on the HBV model forced by today’s climate perturbed with a delta-change from the climate scenarios (Graham 2004). New within ECOSUPPORT was that transient estimates of discharge to the Baltic Sea were made using bias-corrected regional, coupled atmosphere–ocean model outputs and that the Balt-HYPE model provides first process based estimates of how nutrient fluxes to the Baltic Sea may change as a result of future climate change (Arheimer et al. 2012).

### New Generation of Biogeochemical Models for Baltic Sea Management

In 2007 the agreed reductions of the BSAP were evaluated with the Simple As Necessary BAltic Long-Term large-Scale marine biogeochemical model SANBALTS implemented within the decision support system Baltic Nest (Savchuk and Wulff 2007; Wulff et al. 2007). For a contemporary revision of the BSAP in 2013 and the implementation of the Marine Strategy Framework Directive of EU, the BAltic sea Long-Term large-Scale Eutrophication Model (BALTSEM) has been developed as a next generation marine model in the Baltic Nest system (Savchuk et al. 2012). In addition, two three-dimensional Baltic Sea models have been developed further providing additional information on the sub-basin scale (Neumann et al. 2002; Eilola et al. 2009).

### Quality Assurance

Within ECOSUPPORT only models of high-quality were used to produce scenario simulations. The quality assurance followed a common protocol. For instance, Eilola et al. (2011) evaluated and compared individually the hindcast results (1970–2005) from three coupled physical–biogeochemical models relative to the seasonal and annual statistics of salinity, temperature, oxygen, nitrate, ammonium, and phosphate estimated from observations in the Baltic Sea. At representative stations, vertically resolved cost functions were calculated to quantify the biases of model means and standard deviations relative to the corresponding values from observations. Also the pools of nutrients in water and sediment, the extension of hypoxic bottom areas as well as cod reproductive volumes were studied and discussed.

### Multi-model Ensemble Approach

To estimate uncertainties of future projections caused by biases of the global and regional models, natural variability and unknown scenarios of drivers like greenhouse gas and nutrient load emissions and fisheries, a multi-model ensemble approach was applied. The scenarios cover plausible ranges between the most optimistic and pessimistic cases. For instance, one of the nutrient load scenarios is the BSAP. For the marine biogeochemistry more than 50 transient scenario simulations for the period 1960–2100 were performed. The uncertainty is described by the standard deviation among the projections of the ensemble. For details, the reader is referred to Meier et al. (2012b).

### Multi-stressor Approach

The food-web effects of combined multiple-stressors, i.e., climate, nutrient loads and cod fishing, were studied by linking the output from biogeochemical models with an Ecopath with Ecosim (EwE) model for the Central Baltic Sea (BaltProWeb; Tomczak et al. 2012). This model has functional groups from primary producers to seals, including the most important commercial fish of the Baltic, i.e., cod (*Gadus morhua* *callarias*), herring (*Clupea harengus membras*), and sprat (*Sprattus sprattus*). For future projections (2010–2098), the food-web model was driven by two cod fishing scenarios (intensive and low fishing) in combination with environmental forcing (salinity, temperature, hypoxic area, cod reproductive volume and primary production) as projected by the three coupled physical–biogeochemical models in climate and nutrient load scenarios (for details see Niiranen et al. 2013).

### Reconstruction of the Past Baltic Sea Climate Variability

An important aspect of the project was to test the model sensitivity to different drivers on longer time scales by reconstructing the evolution of the Baltic Sea ecosystem from its pristine state around 1850 until today (Gustafsson et al. 2012; Meier et al. 2012c). For this purpose, a key output of the project was to reconstruct the so far longest and most complete set of different forcings from a limited number of available observations. These drivers comprise riverine and atmospheric nutrient inputs (Ruoho-Airola et al. 2012; Savchuk et al. 2012), hydrological data and multivariate daily meteorological fields (Schenk and Zorita 2012). To reconstruct the daily meteorological forcing (High Resolution Atmospheric Forcing Fields, HiResAFF), a nonlinear statistical method was developed. Long historical station records of daily pressure and monthly temperature since 1850 were used to find multivariate analogous target fields within a shorter 50-year regional climate simulation. The advantage of analog-upscaling (Schenk and Zorita 2012) lays in the physical consistency of the reconstructed fields including their regional topographic and variable-specific frequency distributions. As the reconstruction makes use of a limited but relatively constant number of predictor stations, HiResAFF provides a homogeneous long-term reconstruction. Hence, it avoids introducing spurious long-term trends by assimilating different station numbers over time as recently shown for the novel twentieth Century Reanalysis since 1871 (Krueger et al. 2013).

### Policy Dialog with Stakeholders

**Fig. 1:**
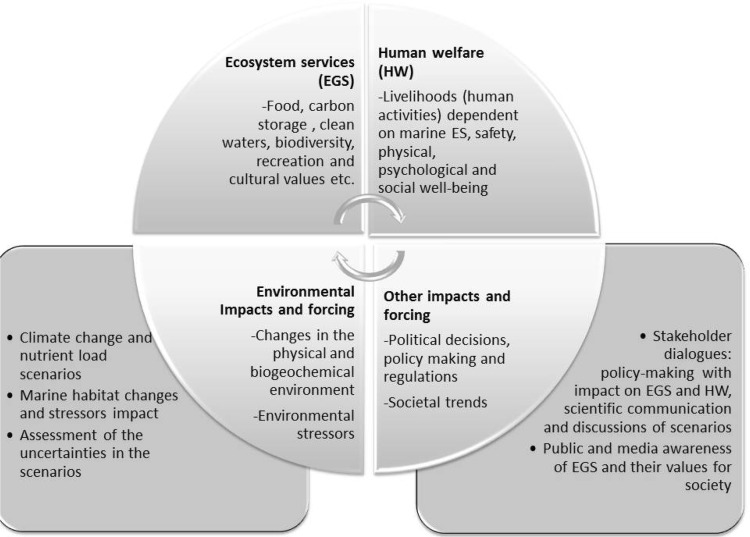
Ecosystem services provide human services. Changes in the physical and bio-geochemical marine environment, as well as policy decisions and societal trends and habits, impact the marine services. ECOSUPPORT aimed at providing awareness and understanding of the human impacts

The anthropogenic pressure on the ecosystem has a direct link to many of the ecosystem services and thereby impact on economic and societal values that provide human welfare (Fig. 1). For example, changes in the marine habitat may change the stock abundance and distribution of both leisure and commercial fish species and the value of coastal recreational activities due to extensive cyanobacteria blooms. Management strategies may have long-lasting consequences for the environment and habits in the society, like the use of fertilizers, increased meat consumption and the use of phosphate in detergents, affect the marine environment considerably. ECOSUPPORT therefore created a communication platform that enabled system understanding, comparison of the outcome of different management scenarios and assessment of the uncertainties involved in modeled projections.

### Geovisualization

Visualization of scientific results has the ability to provide a rapid understanding of complex and heterogeneous data (Tufte 1997; Ware 2004), and several studies have pointed toward the potential increase in engagement and involvement of the audience in participatory events that feature visualization as a methodology (Nicholson-Cole 2005; Salter et al. 2009; Sheppard et al. 2011). Geovisualization takes advantage of human perception capability to find patterns, and allows for large quantities of data and system dynamics to be communicated for a specific spatial context.

**Fig. 2:**
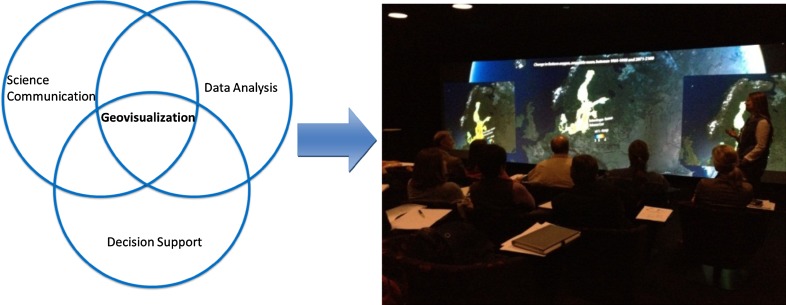
Geovisualization makes use of human perception capabilities when linking large and complex data sets to a geospatial setting. Communication and discussion of ECOSUPPORT scenarios to stakeholders have occurred at a number of occasions, both in the GeoDome and on flat screen. The *photo* is showing discussion and comparison of ECOSUPPORT management scenarios at the Baltadapt workshop, Norrköping 2012

For the purpose of involving stakeholders in a dialog between audience and presenter, modules with visual representations of geospatial data for selected parameters for the Baltic Sea Region were, along with short animations, compiled into the interactive visualization software Uniview^3^ (Fig. 2). The modules were designed to focus on specific issues, including cause, effect and management scenarios for catchment and sea. A narrative was created for each module and the comparative-scenario approach formed a base for further discussions. The interactive presentations were adjusted to suit particular target groups, with the objective to enable data exploration and analysis from different perspectives and to support analytical reasoning and clear understanding of the problems. The interactive application is particularly striking to experience in an immersive GeoDome^4^ environment, which has the potential to further enhance perception and understanding (Neset et al. 2010). ECOSUPPORT therefore used an inflatable, portable GeoDome if feasible (Meier and Andersson 2012), and provided the data material additionally for regular flat screens and through web-access.^5^ An underlying challenge of this format, especially in communication with the general public, was to emphasize the severity of the presented state-of-the-art scenarios, while stressing that management and societal actions are possible and can make a difference, in an effort to avoid creating feelings of hopelessness and apathy of the audience (e.g., Moser and Dilling 2004).

### Challenges and Opportunities in Visualization-Supported Science Communication

To assess the potential of visualization as a tool for science communication and decision support, two separate events were evaluated. Interviews were undertaken with participants of presentations in the GeoDome at the European Strategy for the Baltic Sea Region (EUSBSR) conference in Gdansk 2011. The visualization modules also supported interactive sessions of the BaltAdapt^6^ stakeholder workshop in Norrköping 2012, which were recorded and analyzed as well as evaluated through a survey. BaltAdapt is a transnational flagship project, developing a climate change adaptation strategy for the Baltic Sea region, with focus on the marine and coastal environment. Both events brought together stakeholders from the Baltic Sea Region. The EUSBSR conference featured a broad spectrum of issues and sectors, while the BaltAdapt workshop was focused on climate change and agriculture, with participants representing, e.g., farmer’s associations, agricultural extension services and agricultural administration, from the national, regional, and local level.

## Results

### High-Quality Baltic Sea Models

**Fig. 3:**
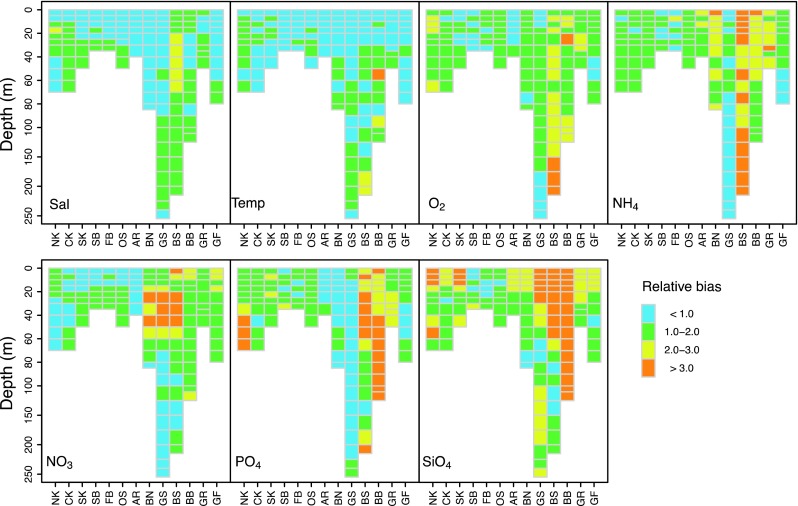
Spatial distribution of the relative bias between simulated and observed dynamics of BALTSEM variables. Comparisons are made for Sal—salinity, Temp—water temperature, and concentrations of O_2_—oxygen, NH_4_—ammonium, NO_3_—nitrate, PO_4_—phosphate, SiO_4_—silicate. At the *x*-axis the following Baltic Sea basins are depicted: NK—Northern Kattegat, CK—Central Kattegat, SK—Southern Kattegat, SB—Samsø Belt, FB—Fehmarn Belt, OS—Öresund, AR—Arkona Basin, BN—Bornholm Basin, GS—Gotland Sea, BS—Bothnian Sea, BB—Bothnian Bay, GR—Gulf of Riga, GF—Gulf of Finland

The extensive and detailed model-data comparison for the period 1970–2006 showed that biogeochemical models were capable to reproduce much of the nutrient biogeochemical cycling in the Baltic Sea (Eilola et al. 2011). For example, the Nest component model BALTSEM simulated successfully both the inter-basin spatial gradients and temporal variations at seasonal to long-term scales (Fig. 3; Savchuk et al. 2012). None of the three models was perfect in all aspects. The ensemble means matched the data better than, or as good as, the results of any of the individual models (Eilola et al. 2011). The evaluation of model’s performance revealed that, in addition to a number of model-specific needs for improvement, there are a few major model-data discrepancies like (1) the large model-data biases in the Gulf of Bothnia, especially in the Bothnian Bay; (2) the markedly lower rates of primary production compared to those reported from observations; (3) the significant differences in the nutrient turnover time scales between the models, caused primarily by differences in simulated sediment nutrient pools and fluxes.

### Projected Changes in the Regional Climate System

According to Meier et al. (2012c) water temperature in the Baltic Sea at the end of the twenty-first century will be higher and salinity and oxygen concentrations will be lower than any values since 1850. Although changes in the Baltic Sea water balance suffer considerably from model uncertainties (Meier et al. 2006), the seasonal dynamics of discharge to the Baltic Sea are expected to increase compared to today’s pattern for all ECOSUPPORT projections (Fig. 2 in Meier et al. 2012b). In general, winter discharge to the Baltic Sea increases while summer discharge decreases. The spring flood peak is reduced for all scenarios. It was much more difficult to detect trends in overall nutrient fluxes to the Baltic Sea as a result of a future climate. Interannual variation was much larger than an eventual long-term trend for each climate projection. Nevertheless, there was a consistent change to the seasonal distribution of nutrient concentrations to the Baltic Sea (Arheimer et al. 2012). The seasonal variations of both nitrogen and phosphorous concentrations were dampened with consistent decreases in winter concentrations seen. For phosphorous, there is also an indication that summer concentrations may increase and that more frequent short-term peaks in concentration may occur. The experiments with combinations of remedial measures and climate change in the Balt-HYPE model indicate that there is a possibility to reach all the BSAP targets in the future for most marine basins by the end of this century. For N, the impact of climate change is of the same order as the expected reduction from remedial measures, according to the results of the model experiment. Further, there is a higher probability to reach BSAP targets for P than for N. Thus, climate effects need to be accounted for when estimating the long-term effects of the BSAP.

The future marine ecosystem in the Baltic Sea was projected to change unprecedentedly compared to the past 150 years, and nutrient load reductions and sustainable fishery may be even more important in the future to ensure a healthy marine ecosystem when the stresses from climate change increase (Meier et al. 2012a, b, c; Niiranen et al. 2013). Applying various nutrient load scenarios, it was also shown that under the impact of warming climate hypoxic and anoxic areas will very likely increase or at best only slightly decrease (in case of optimistic nutrient load reduction scenarios) compared to present conditions, regardless of the used global model and climate scenario (Meier et al. 2011b). For the end of the century, prolonged growth and a more than twofold increase in the mean cyanobacteria biomass and nitrogen fixation was found using a coupled biological–physical model with an advanced cyanobacteria life cycle model (Hense et al. 2013). In addition, considerable changes in the spring bloom at least in the northern Baltic Sea are expected as a consequence of the shrinking ice cover in warmer climate (Eilola et al. 2013).

The combination of regional drivers, i.e., cod fishing and nutrient loads, had a large effect on the projected futures of the Central Baltic Sea food-web. In the worst-case scenario, i.e., high cod fishing and nutrient loads a eutrophied and strongly sprat-dominated ecosystem was projected, while the best-case scenario resulted in a cod-dominated ecosystem with eutrophication levels close to present. However, the regional management decisions were not fully able to compensate for some directional climate change effects. For example, cod was negatively affected by worsening reproduction conditions, due to decreasing salinities, and its biomass was projected to decrease during the second half of the twenty-first century across all combinations of fishing and nutrient load scenarios. This indicates that only reduced fishing should be permitted under future climate conditions, if moderate to high cod biomasses are desired (Lindegren et al. 2010). In general, top predatory fish cod was mainly affected by changes in fishing mortality, while phytoplankton and several zooplankton groups responded almost solely to changes in climate and nutrient conditions. The intermediate trophic level groups, i.e., sprat, herring, and *Pseudocalanus acuspes* were most affected by the combination of top-down (cod fishing) and bottom-up forces (nutrient loads and climate drivers). These groups are suggested to have an ecosystem structuring role in the Baltic Sea (Möllmann et al. 2009), indicating the importance of evaluating the interplay of multiple driver effects when projecting the future of the Baltic Sea food-web.

### Uncertainties in Projected Biogeochemical Cycles

According to Meier et al. (2011b), uncertainties in projected biogeochemical cycles are dominated by unknown future nutrient loads, biases of the GCMs and biases of the biogeochemical models. We found largely differing sensitivities of the models to changing nutrient loads. Nevertheless, all biogeochemical models suggest that the BSAP will be less effective in future climate than in present climate.

### Detection and Attribution in Physical and Biogeochemical Variables

**Fig. 4:**
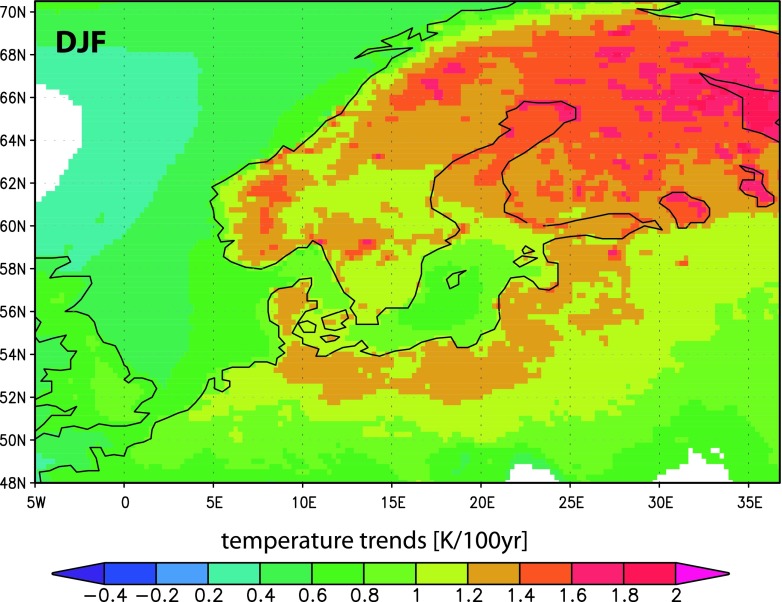
Seasonal near-surface temperature trends in winter 1850–2009 (HiResAFF). Non-significant trends (*p* < 0.05) in *white*

With exception of the somewhat underestimated warming trends in spring, HiResAFF clearly reflect the observed strong seasonal warming trends since 1850. As shown in Gustafsson et al. (2012), the reconstructed trends closely match those derived from coarsely gridded observations like CRUTEM3 over the Baltic Sea since 1871. Besides a high spatial resolution of ~25 km, a novelty of HiResAFF relates to the physically consistent reconstruction of near-surface conditions also over sea-areas where usually little or no observations exist back in time. As an example, Fig. 4 shows smaller winter warming trends over deeper sea-areas like the Baltic Proper.

### Impacts of ECOSUPPORT Results on Management

**Fig. 5:**
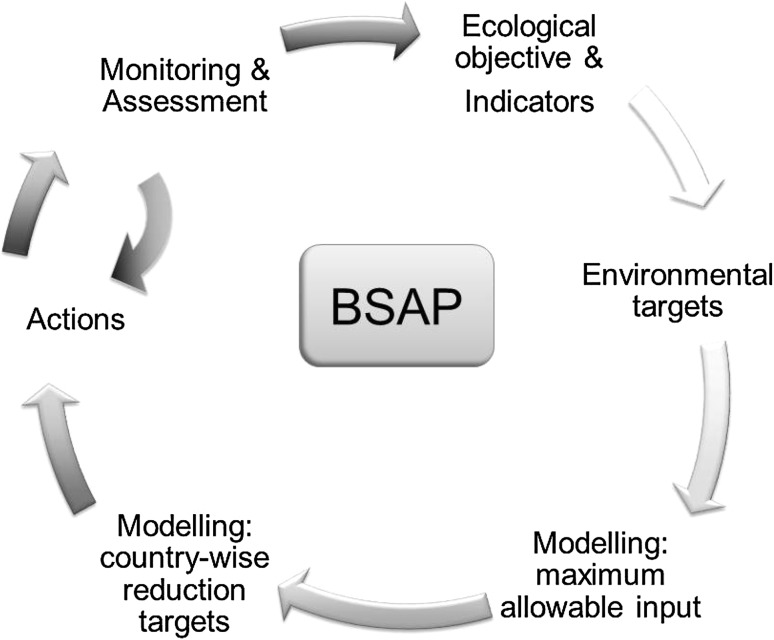
The work process of the Baltic Sea Action Plan

HELCOM’s BSAP has been further developed since 2007 and now comprises a number of steps (Fig. 5). The basis of the BSAP has been the definitions of the ecological objectives, agreed upon as the vision of an environmentally healthy Baltic Sea, for example clear water and end to excessive algal blooms. Indicators were developed that would reflect the objectives. Monitoring enables the assessment of the current environmental status, as reflected by the indicators. Quantitative target indicator values for a good environmental status are also established, primarily based on monitoring data and statistical analysis. In the following step, the relationships between pressure (i.e., nutrient loads) and target variables are quantified by means of physical–biogeochemical modeling. The pressure–response relationships differ for the various regions within the Baltic Sea because of differences in, e.g., circulation, ecosystem and nutrient loads. The results of the modeling are basin-wise MAIs of nutrients that will result in a development toward eventually reaching the targets. The MAIs of nutrients, as a first step toward implementation, are allocated as country-wise reduction target where the necessary load reductions are distributed by basin to the contracting countries according to polluter pays principles, and what is considered fair burden and is in agreement with the BSAP. The implementation of nutrient load reductions is planned through national implementation plans.

Leading up to a Ministerial Meeting in 2013, a review of the BSAP agreement from 2007 with its preliminary nutrient reduction targets has significantly gained from ECOSUPPORT developments, e.g., the determination of targets within the TARGREV project (Review of the ecological targets for eutrophication of the HELCOM BSAP, see HELCOM 2013) was improved by using results from the long-term hindcast reconstruction 1850–2007 (Gustafsson et al. 2012). From the ensemble mean of the historical simulations guiding targets for nutrients and chlorophyll *a* were derived by estimating indicator levels around 1900 with the levels from the 1970s. Further, the MAI calculation has been strengthened by multi-model validation studies (Eilola et al. 2011) and model developments leading to a new generation of high-quality Baltic Sea models (Savchuk et al. 2012). Although the sensitivity of the models to nutrient load changes largely differed, all models showed the same response qualitatively (Meier et al. 2012b). HELCOM ministerial meetings, both in 2007 and 2010, noted that climate change will have impacts and this should ultimately be reflected in HELCOM policies. Specifically, both climate change aspects as well as ensemble modeling should be reflected in the reviewed BSAP at the 2013 meeting.^7^ The HELCOM Executive Secretary emphasized the usefulness of ECOSUPPORT results in the review of the eutrophication segment of the BSAP.^8^ Hence, future revisions of the BSAP will hopefully include both changing climate and ensemble modeling to estimate uncertainties of projections.

For a comprehensive summary of ECOSUPPORT results the reader is referred to the AMBIO Special Issue: ECOSUPPORT—Different Ecosystem Drivers Under Future Climate Scenarios in the Baltic Sea (Reckermann 2012 9).

## Discussion

The ECOSUPPORT results indicate that it is very likely that the work with improving the health of the Baltic Sea will take place in a transient Baltic Sea. As the nutrient reductions will not be as efficient in a future climate, it is important to assess how much additional reductions need to be accomplished in order to reach the goals of BSAP in a changing climate. Further, it is important to understand “when to stop” since improvements in the environment will continue long after the actual reduction took place. The work will undoubtedly be afflicted with uncertainties arising from different sources. The climate change scenarios are uncertain and reflect merely the present state-of-the-art knowledge, and will have to be revised to consider new development, mitigation strategies and technology. The management scenarios are uncertain in a changing climate since both needed reductions and catchment loads are uncertain: warming will, for example, induce higher evaporation and mineralization rates with impact on soil processes and increased precipitation changes in runoff rates, annual cycles and flooding. The processes are considered in the Balt-HYPE model, but process knowledge needs to be further improved (Arheimer et al. 2012). Furthermore, the catchment will undergo changes due to changes in agricultural practices, improved technology for land nutrient retention, changing vegetation in a warmer and dryer/wetter climate, demographic changes, etc.—effects not taken into account in the present scenario narrative. Present scenarios also lack understanding of realistic present and future atmospheric deposition of nutrients. When it comes to ecosystem structure and functioning, we will move into a Baltic Sea state where the food-web models cannot be evaluated with present state observation and understanding since changes in, e.g., ocean acidification, lower salinity and impact on invasive species will bring a state unknown to the research community (Niiranen et al. 2012). This can have implications for management actions to protect marine areas and restrict fisheries, but is difficult to foresee with state-of-the-art models and understanding. The ECOSUPPORT efforts not only detected model deficiencies, e.g., for the biogeochemistry in the northern Baltic Sea, which needs to be improved, but also differences in model behavior with implications for management. This relates, e.g., to the changes of the internal loads (nutrient pools) under the nutrient load reduction scenario, which behave differently in the different Baltic Sea models and therefore the models have different response time between abatement and improvement.

There is a need to develop modeling strategies to support adaptive management under combined pressures. ECOSUPPORT was a pioneering effort showing the way and illustrating challenges and opportunities for time-dependent adaptive management. The way to approach the future and handle the uncertainties may be to make the BSAP process operational (Fig. 5). Also in the future the focus will have to be on the monitoring and assessment, in order to evaluate the ecosystem that changes as a response to applied abatement strategies. Models and scenarios should be continuously updated with state-of-the-art understanding and a multi-model, multi-scenario approach (ensemble modeling) would be preferable in order to take uncertainties into account (Meier et al. 2012c). The MAIs and reduction targets will have to be revised to take changes in the physical environment into account. The ECOSUPPORT projections also indicate future regional differences, e.g., the warming over the northern region will be more pronounced than over the southern region, and river runoff is indicated to increase in the northern region and maybe even decrease in southern areas. These changes mean that geographical areas of the Baltic Sea will have different salinity, temperature, and stratification in future compared to present climate. Hence, ecological objectives can either be more easy or difficult to reach than today. This indicates that the country allocations might also have to be changed due to achieved abatements and environmental targets, and should be adjusted to give maximum effect in the most cost-efficient way.

Although ecosystem services and climate change are phenomena that can be defined and quantified using a strictly scientific approach, the decision-making about them is a social process, where scientific information might be of secondary importance. Education and information campaigns could be an entry point to raise public awareness and inform managing strategies and coastal planning.

Although ECOSUPPORT results are highly relevant for marine management, some shortcomings of the approach and future research needs were identified:

1. To improve the simulation of biogeochemical cycling, especially in the northern Baltic Sea, existing models should be extended with carbon cycling including dissolved organic carbon, nitrogen, and phosphorus (DOC, DON, and DOP).
2. Nutrient retention in the coastal zone is poorly understood. The coastal zone filtering effect should be studied using high-resolution modeling of the coastal ecosystem.
3. Models for lower and higher trophic levels should be two-way coupled to study bottom-up and top-down controls of the marine ecosystem.
4. To better quantify the carbon and nutrient inputs from land, modeling of the land-sea continuum needs to be further improved, e.g., by including the interactions between climate, land use and socio-economy.
5. For a comprehensive risk assessment the multi-stressor approach should be extended and hazardous substances and invasive species should also be taken into account.

## Footnotes

1
http://www.baltex-research.eu/ecosupport; http://link.springer.com/journal/13280/41/6; http://www.baltex-research.eu/ecosupport/publications.html.

2 Coupled Model Intercomparison Project Phase 5, see http://cmip-pcmdi.llnl.gov/.

3
http://www.scalingtheuniverse.com.

4
http://geodome.info.

5
http://www.baltex-research.eu/ecosupport/dss/index.html.

6
http://www.baltadapt.eu.

7
http://www.helcom.fi/press_office/news_helcom/2013/en_GB/Climatews_Feb13/.

8
http://www.bonusportal.org/news_room/news_archive/minister_ek_visits_the_bonus_offices_br_bonus_a_good_example_of_successful_cooperation.685.news.

9
http://link.springer.com/journal/13280/41/6/page/1.
